# Understanding scientific progress: the noetic account

**DOI:** 10.1007/s11229-021-03289-z

**Published:** 2021-07-14

**Authors:** Finnur Dellsén

**Affiliations:** 1 Faculty of History and Philosophy, University of Iceland, 101 Reykjavík, Iceland; 2 Department of Philosophy, Law, and International Studies, Inland Norway University of Applied Sciences, 2624 Lillehammer, Norway

**Keywords:** Scientific progress, The noetic account, Understanding, Dependence relations, Truthlikeness, Knowledge

## Abstract

What is scientific progress? This paper advances an interpretation of this question, and an account that serves to answer it (thus interpreted). Roughly, the question is here understood to concern what type of cognitive change with respect to a topic *X* constitutes a scientific improvement (to a greater or lesser extent) with respect to *X*. The answer explored in the paper is that the requisite type of cognitive change occurs when scientific results are made publicly available so as to make it possible for anyone to increase their understanding of *X*. This account is briefly compared to two rival accounts of scientific progress, based respectively on increasing truthlikeness and accumulating knowledge, and is argued to be preferable to both.

## Introduction

The progress of science is astounding. Just two centuries ago, people suffering from infectious diseases would have been told that their illnesses were caused by ‘miasma’, i.e. impure air arising from decomposing organic matter. Progress was made in the late 19th century, when the miasma theory was replaced by the theory that infectious diseases are caused by unobservably small entities passing between organisms, i.e. ‘germs’. The progress that has since been made builds on this theory, e.g. in the discovery that some infectious diseases (such as tuberculosis and the plague) are caused by bacteria, while others (such as seasonal influenza and COVID-19) are caused by viruses. So at least on the topic of infectious diseases, scientists have made significant progress over the years. But why? In virtue of what do these developments count as progressive? *What is scientific progress?*

It is natural to worry that this question is too ‘philosophical’, in the pejorative sense of the term, to admit of a definitive answer. For example, Chang describes it as “one of the most significant issues in the philosophy of science today”, but then immediately notes its “immense difficulty” (Chang 2007, p. 1). Part of that difficulty is surely that the question itself can seem *unclear*, *misguided*, and even *pointless*: (i) What would it even be to advance a philosophical account of scientific progress? (ii) Doesn’t science progress in a variety of quite different ways, depending on the scientific field, its methodology, or even the particular research project in question? (iii) And even supposing that some general account of scientific progress could be provided, what would be the point of such an exercise?

In this paper, my first aim is to show that these worries can be convincingly allayed. In response to (i), I will argue that the question, ‘What is scientific progress?’, has at least one interpretation on which the question itself is perfectly clear and intelligible. In response to (ii), I will argue that, on this interpretation, there is no particular reason to think that a general account of scientific progress cannot be provided. Finally, in response to (iii), I will argue that this interpretation of the question makes evident why answering the question thus interpreted is important—viz., not just because of its intrinsic intellectual importance, but also due to the practical implications of different answers.

My second main aim for this paper is to elaborate and argue for a particular answer to the question thus interpreted. This answer is based on the idea that progress regarding some phenomenon consists in increasing our potential to *understand* that phenomenon, a proposal closely akin to what I have previously called *the noetic account* of scientific progress (Dellsén 2016). The current paper develops this proposal by coupling it with a general definition of ‘understanding’, and by specifying whose potential increase in understanding is at issue. The resulting account is then compared to two rival accounts, which respectively define progress in terms of increasing truthlikeness and accumulating knowledge, and defended against three potential objections.

## The question of scientific progress

As promised in the introduction, I start by clarifying the question at issue, ‘What is scientific progress?’, so as to make clear why it’s intelligible, tractable, and important. I will proceed by first making a number of preliminary points to precisify the relevant concept of scientific progress, before then returning to the question itself, how to go about answering it, and why that matters.

A first thing to note is that *scientific progress*, in contrast to *scientific change*, is a partly normative or evaluative concept, i.e. a ‘thick’ concept. To say that science made progress between $t_1$ and $t_2$ is to say that there was some improvement in or of science between $t_1$ and $t_2$ (cf. Niiniluoto 2019, §2.2). This is not to say that the overall state of the world is better at $t_2$ than $t_1$, of course, since other things might have changed for the worse between $t_1$ and $t_2$. Nevertheless, something must improve in order for scientific progress to occur. It follows immediately that choosing between accounts of scientific progress has normative implications. For example, all else being equal, if one account implies that successfully completing a research project would achieve scientific progress while another account implies that no progress would be made even on a successful completion of that project, then the first account, but not the second, implies that scientists have some reason to pursue that project.

This in turn has important implications for the methodology most appropriate in debates about scientific progress. Specifically, this arguably means that accounts of scientific progress should be tested against our considered normative judgments, e.g. regarding whether to pursue some research project at the expense of another. By contrast, these accounts should not be tested against our linguistic intuitions about whether we would initially and unreflectively be inclined to refer to a given episode as ‘scientific progress’. After all, if it turned out that the term ‘scientific progress’, as it is in fact used in natural language, systematically classifies less pursuitworthy research projects as more ‘progressive’ (and vice versa), then we should surely modify, or re-engineer, the concept of scientific progress so as to fit with our considered judgments about what sorts of pursuits are in fact most valuable.^1^

Second, what is the ‘something’ that needs to improve between $t_1$ and $t_2$ in order for scientific progress to occur during this period? It’s tempting to answer that it is science as a whole, or perhaps some particular scientific discipline. But for reasons noted by Niiniluoto (2019, §2.1), accounts of scientific progress are not meant to cover all ways in which a scientific discipline could improve. For example, a discipline could improve by virtue of receiving more funding, by increasing its independence from pernicious outside influences, or by increasing gender equality among scientists. Although such changes would clearly be *improvements* in a general sense, the debate about scientific progress concerns a narrower class of changes that Niiniluoto labels ‘cognitive’. Dellsén (2018, p. 2) characterizes these as having “to do with improvement in our theories, hypotheses, or other representations of the world, rather than other improvements of or within science”.

Third, some recent discussions of scientific progress have introduced a useful distinction between what constitutes scientific progress and what merely promotes it (see, e.g., Bird 2008, p. 280; Dellsén 2016, p. 73). A cognitive change *constitutes* progress when the change is an improvement in some respect regardless of what other changes are thereby brought about, or made more likely to be brought about, at some later time. By contrast, a cognitive change *promotes* progress when the change is an improvement only in so far as later changes are brought about, or made more likely to be brought about (i.e. probabilified), by that cognitive change. Both constituting and promoting progress thus count as improvements, but the latter counts as an improvement only in virtue of leading to or probabilifying an occurrence of the former at some later time.^2^ For example, consider the formulation of a new concept that is subsequently used to state a theory which, let’s suppose, is an improvement on the previous theories in some domain. The formulation of this concept would arguably not itself constitute progress, but it would definitely promote progress in so far as it helps scientists to state, and thus eventually accept, a progressive theory.^3^

It should be clear that, depending on the the phenomenon in question, a number of quite different things might promote progress to a greater or lesser extent. For example, randomized controlled trials promote progress on the effectiveness of medical treatments, while computer simulation models promote progress on biological and economic systems (and not normally vice versa). As this makes clear, there is no reason to think there is a unified general story to tell about what promotes progress across all scientific disciplines. Indeed, what presently promotes progress within some discipline might cease to do so, or do so to a lesser extent, in the future, given technological or methodological changes. So there is a strong case to be made for a kind of ‘pluralism’ about what promotes progress. Note, however, that it does not follow that we should be pluralists about what constitutes progress, since these different ways of promoting progress might well be instrumental for achieving the same type of cognitive improvement.

Fourth, scientific progress is gradable—a matter of degree—in the sense that a given episode can be more or less progressive, perhaps in addition to being progressive outright (in a binary sense).^4^ Although some theorists fail to address what determines degrees of progress, instead providing accounts only of outright progress,^5^ this is arguably an unmotivated restriction of the topic at hand. If at all possible, an account of scientific progress should explain not just why a given episode is progressive, but also why it is more or less progressive than another episode—at least when the two episodes concern the same topic. For example, an account of scientific progress worth its salt should explain why adopting Tycho Brahe’s geo-heliocentric model of the solar system would not have constituted as much progress as adopting Kepler’s version of Copernicus’s heliocentric model, even though adopting either model would arguably have been an improvement on the earlier Ptolemaic model.

A fifth and final point is that we can distinguish between a topic-specific concept of scientific progress (*progress-on*-*X*), and a more general, across-topic concept of scientific progress (*overall progress*). Consider an episode that exhibits cognitive improvement with respect to one topic $X_1$, and yet simultaneously exhibits the opposite, i.e. cognitive decline, with respect to another topic $X_2$. How should we describe such an episode in terms of scientific progress? Well, if we are using a topic-specific concept of scientific progress, *progress-on-X*, such an episode can simply be described as simultaneously exhibiting progress on $X_1$, and the opposite of progress, i.e. regress, on $X_2$. On the other hand, if we are using the general, across-topic concept of *overall progress*, then the question of whether there is progress in that sense during the episode presumably turns on whether the there was enough progress made on $X_1$ to outweigh the regress on $X_2$. This suggests that *overall progress* can be defined as the aggregation or sum of degrees of progress (and regress) on the various different topics $X_1$, $X_2$, etc., on which cognitive change takes place during an episode.^6^ Since progress-on-*X* therefore seems to be the more fundamental notion of the two, we will primarily be concerned with it in what follows.

To summarize, then: scientific progress is a type of *improvement* over time, so characterizing a change as progressive has immediate normative implications; this improvement concerns *cognitive* changes specifically, rather than other types of improvements in or of science; the question as to what *constitutes* such progress can, and should, be distinguished from what *promotes* it; scientific progress is *gradable*, in the sense that an episode can be said to be more or less progressive in addition to being outright progressive; and finally, a concept of *progress-on*-*X* can be distinguished from, and yet used to define, a concept of *overall progress*. With all this in mind, we can say that the seemingly simple question ‘What is scientific progress?’ can be precisified as follows:

(Q) What type of cognitive change with respect to a given topic *X* constitutes a (greater or lesser degree of) scientific improvement with respect to *X*?

In the introduction, I mentioned three types of worries about our original question, ‘What is scientific progress?’. The first was that the question was unclear. That, I submit, need no longer worry us, since the original question can now be replaced by the painstakingly precise (Q). The second worry was that science clearly progresses in different ways, depending on the scientific field, methodology, or research project. That worry is assuaged by pointing out that different disciplines may promote progress in different ways even if the same type of cognitive change constitutes progress across those disciplines. Furthermore, by relativizing progress to a particular topic *X*, we have opened up the possibility that what constitutes progress with regard to one topic $X_1$ could differ from what constitutes progress with regard to another topic $X_2$. Finally, the worry that an account of scientific progress would be pointless is clearly misconceived, since as we have seen the question of progress is ultimately a normative question that has direct implications for what scientists ought to spend their time and resources on. It is thus an issue that is of obvious relevance not only to philosophers of science, but also to science administrators and working scientists.

## The noetic account, revised and elaborated

In this section, I develop an account of scientific progress, i.e. an answer to (Q), that centers around the concept of *understanding*. To a first approximation, the account holds that scientific progress with respect to *X* consists in a change in the publicly available information about *X* that helps us increase our understanding of *X*, where ‘increased understanding’ is defined as gaining a more accurate or comprehensive model of *X*’s dependence relations, such as its causal relations. In so far as such dependence relations ground explanation and prediction, e.g. through causal explanation, this account implies a strong link between scientific progress, on the one hand, and explanation and prediction, on the other. In this respect, the current account resembles the original noetic account (Dellsén 2016). Indeed, although there are differences between the two accounts—some of which will be brought out below—the former is sufficiently close to the latter to be viewed as a modification and elaboration of the original account. In what follows, I spell out this new version of the noetic account.

Let me first make a methodological point. In what follows, I offer a definition of the the relevant notion of understanding, before defining progress in terms of that notion. Although I believe that this definition of understanding is at least as good as any alternative definition on offer,^7^ I will not provide any arguments to that effect in what follows. Indeed, those who prefer a different definition of the concept of understanding may take the definition that I offer here as purely stipulative. After all, our concern is ultimately not with the nature of understanding as such, but rather with an account of scientific progress—i.e., with answering (Q). For those purposes it is merely a matter of terminology whether the account is stated in terms of the notion of understanding or in terms of the concepts used below to attempt to define that notion. Thus, if you prefer, you may take ‘understanding’ to be a mere label for the cognitive state, described below, in terms of which the current noetic account defines scientific progress.

### Dependency models

The definition of understanding to which I appeal in what follows makes use of the notion of a *dependency model*. This is a model of the dependence relations that aspects of a given phenomenon stand in, or fail to stand in, to other aspects of the phenomenon, or of other phenomena. Such a model thus contains information about relations between (aspects of) phenomena—both ‘positive’ information about how they are related, and ‘negative’ information about how they fail to be related. The relations in question are *dependence relations*, the paradigmatic instance of which is *causation*, but which may include other dependence relations such as *grounding*. The relata of these relations are variables, rather than specific or actual values of such variables; they may be either continuous (e.g., an object’s mass *m*) or discreet (e.g., a population size *N*). Thus dependence relations encode information not just about the actual state of some phenomenon, but also how the phenomenon would have been different if other things had been different in some specific way.

So a dependency model of a phenomenon, in so far as it is accurate and comprehensive, encodes information about dependencies. Most dependency models that are even just somewhat accurate and comprehensive will be enormously complex, but let me illustrate with a simple, toy example. According to Hooke’s law, the force exerted by a spring on an object fastened to it, displaced at a distance *x* from a relaxed position, is given by $F_s=-kx$, where *k* is a positive constant specifying the ‘stiffness’ of the spring (the minus sign indicates that that the force $F_s$ is opposite to that of the displacement *x*). So if the object is pulled a distance *x* and then released, then assuming as an idealization that no other forces act on the object, the force $F_s$ will accelerate the object towards its relaxed position in accordance with Newton’s second law, $F=ma$. Hence the object’s acceleration when released will be $a=-\frac{kx}{m}$. This tells us a great deal about what the object’s acceleration depends on, e.g. its mass; and, indeed, about what it does *not* depend on, e.g. its volume. This is a paradigmatic example of a (very simple) dependency model, in this case of a composite phenomenon consisting of an elastic spring and an object attached to it.

In the above example, all the dependence relations involved are causal—at least arguably so. As I have intimated, however, this is not always the case. Suppose we supplement this model with information about how the spring’s stiffness *k* is determined. Now, *k* can clearly be *calculated* from Hooke’s law by plugging in actual values for the force $F_s$ and distance *x*. But what *k* *depends on* has to do with various facts about the spring itself, e.g. its length at relaxed position, the number of coils, the diameters of those coils, and the material from which the spring is constructed. The relation between *k* and these facts about the spring is arguably not causation; rather, it is something closer to grounding.^8^ So a more comprehensive dependency model of the spring and its attached object includes these arguably-non-causal dependence relations as well. An even more comprehensive dependency model would contain even more (causal or non-causal) information of this sort.

A more *accurate* dependency model, by contrast, would correct some of the inaccuracies contained in the above model. For example, it is of course not true of any real system of this sort that the *only* force that acts on the object is due to the spring. Hence we cannot really identify the *F* in $F=ma$ with the $F_s$ in $F_s=-kx$, as would strictly speaking be required to derive $a=-\frac{kx}{m}$. For example, if the spring and object are located on an horizontal surface, then a (non-zero) friction force, $F_f = -\mu mg$, will act against $F_s$, so that $F=F_s-F_f$. From this it follows immediately that $a = \frac{F}{m} = -\frac{kx}{m}+\mu g$. We thus have a more accurate dependency model of the spring and the attached object, e.g. in that we see that (and how) the acceleration of the object depends on the friction between the object and the surface. This model tells us, among other things, that the effect of friction on acceleration does not depend on the object’s mass.

### Scientific understanding

So much for dependency models. What has this got to do with understanding? Well, on the view of scientific understanding I favor (Dellsén 2020), the latter can be defined in terms of the former: an agent *S* *understands* *X* if and only if *S* grasps a sufficiently accurate and comprehensive dependency model of *X*; and *S*’s *degree of understanding* of *X* is proportional to the accuracy and comprehensiveness of their dependency model of *X*. I note immediately that the target of this type of understanding, *X*, is some part of our world; not a mere representation thereof, such as a theory, concept, or explanation. In the literature on understanding, this type of understanding is generally referred to as ‘objectual understanding’, and often contrasted with ‘understanding why’ or ‘explanatory understanding’ (see, e.g., Kvanvig 2003; Khalifa 2013; Kelp 2015). I note also that there are many terms that intentionally do not occur in this definition, including notably ‘justified’ and ‘know’. As we shall see, understanding can come apart from what philosophers typically mean by those terms.

What about *truth*? Well, it follows immediately from the definition above that an increased understanding of *X* can be identified with having a more accurate, or more comprehensive, dependency model of *X*. Thus incorporating true information into one’s dependency model of *X*, in so far as it reveals something about the relevant dependence relations, will necessarily increase one’s understanding of *X*. In this sense, understanding is ‘factive’. And yet the current definition allows for departures from the truth to increase understanding, most straightforwardly since incorporating an intentional approximation, which deliberately contains a slight falsehood, can significantly increase the comprehensiveness of a model at the expense of a small loss of accuracy. To return to our earlier example, setting $F=F_s$—although strictly false, since $F_f$ is non-zero—initially contributed to understanding the object’s acceleration. However, as the subsequently modified version of the example also illustrates, we would in that case gain even more understanding by de-approximating and instead setting $F=F_s-F_f$.

Many theorists associate understanding very closely with explanation (e.g., Strevens 2013; de Regt 2017; Khalifa 2017). On the above definition, this is correct only in so far as understanding consists in modelling the dependence relations that form the ontological basis for explanation. Thus it is true that, when it’s possible to explain *X* or aspects of *X*, a completely accurate and comprehensive understanding of *X* will provide all the information needed for explanation. However, understanding can also consist in the realization that a phenomenon or some aspect thereof cannot be explained at all, or that it cannot be explained by some particular other phenomenon or aspect thereof. For example, we noted before that the decrease of acceleration due to friction exerted on an object moving on a horizontal surface does not depend on its mass; hence it cannot be explained by its mass. Nevertheless, incorporating this very fact—that the decreased acceleration due to friction does not depend on the object’s mass—into our dependency model increases our understanding of the object’s acceleration. So understanding, by the above definition, is in this way a more general concept than explanation, and should not simply be identified with the cognitive benefits of explanation.

Related to this is the fact that understanding brings with it various other cognitive benefits. Chief among these are manipulation and prediction. Consider the spring again. Suppose you want to modify the surface on which the object is placed so as to make sure that it does not move at all when released at a distance *x* from the spring’s relaxed position. You might do this by replacing a smooth surface with one that is covered in sandpaper, for example. If you grasp the final dependency model described above, in which $a=-\frac{kx}{m}+\mu g$, this can be achieved by setting $a=0$ and then isolating the friction coefficient $\mu = \frac{kx}{mg}$, which measures the extent to which the object and the surface create friction with one another. This tells you what grit size you need for the sandpaper, for example, so as to get the object to stay put at a given distance *x* from a relaxed position. Similarly, for the purposes of prediction, you need to know what *will* happen given the current state of the spring—or what *would* happen given some counterfactual state of the spring. Your understanding, via your dependency model, tells you precisely that, e.g. by revealing what the acceleration of the object will be when released at distance *x*, or would be if released at some alternative distance $x'$.

### Scientific progress

Now, how do we get from this definition of understanding to an account of scientific progress, i.e. to an answer to (Q)? We might say that scientific progress, i.e. the type of cognitive change with respect to a given phenomenon *X* that constitutes scientific improvement relative to *X*, is *increased understanding* of *X*. However, this is incomplete as it stands, since it fails to specify *whose* understanding increases in scientifically progressive episodes. Indeed, there is a more general question in the vicinity here that applies to any account of scientific progress, viz. *whose* cognitive attitudes must in some way improve in order for scientific progress to take place? In so far as this question has been discussed at all, the agents in question have been assumed to be the relevant scientists themselves, either individually or collectively as a group.^9^ Applied to an understanding-based account, this implies that scientific progress relative to *X* occurs precisely when scientists themselves (individually or collectively) increase their understanding of *X*.

On further reflection, however, this exclusive focus on the cognitive attitudes of scientists themselves seems unmotivated. If all that really improved through scientifically progressive episodes were the scientists’ own attitudes, e.g. in increasing their understanding, then why should non-scientists care about scientific progress at all? In particular, how could the extensive funding of ‘pure’ scientific research, with no clear practical benefits for non-scientists, be justified if scientific progress merely consisted in some scientists improving *their* cognitive attitudes? In light of this problem, I suggest we move to a conception of scientific progress according to which it is not the cognitive attitudes of those who make scientific progress that determine whether an episode is progressive; rather, progress is determined by the publicly available information, such as that contained in peer-reviewed journal articles, on the basis of which any relevant member of society (including scientists but not excluding non-scientists) can form or sustain the relevant type of cognitive attitudes. In the case of the noetic account, then, I suggest that what matters to progress on *X* is whether changes in the publicly available scientific information makes it possible for relevant members of society to increase their understanding of *X*.^10^

We are now—finally!—in a position to formulate a revised noetic account of scientific progress:

**The noetic account (restated):** The type of cognitive change with respect to a given phenomenon *X* that constitutes (a greater or lesser degree of) scientific improvement relative to *X* is a change due to scientific research in the publicly available information that enables relevant members of society to increase their understanding of *X*.

This somewhat Procrustean formulation of the account is meant to explicitly mirror the question to which it is an answer, (Q). More colloquially, the noetic account thus reformulated holds that scientific progress consists in making available scientific information that helps us as a society to better understand relevant phenomena. Given the identification of understanding with dependency modelling, scientific progress enables us to model dependencies in these and related phenomena—which, in turn, helps us explain, manipulate and predict them on a regular basis.

At this point it is worth reiterating that there may be many different ways of *promoting* scientific progress even if there is a single type of cognitive change that *constitutes* progress (see Sect. 2). On the noetic account, progress is promoted by any development that leads to or probabilifies changes in available scientific information which enable relevant members of society to increase their understanding. Thus most of the everyday activities of working scientists—including, for example, experimentation and observation, theoretical exploration, and developing novel methods—will promote scientific progress on the noetic account, because and in so far as these are important steps towards enabling us to increase our understanding of some phenomena. To say that these activities promote progress is emphatically not to say that they are less important than the activities that constitute progress. After all, a given episode (e.g. an especially decisive experiment) might promote a great deal more progress than another episode (e.g. a minor modification to a causal model) constitutes, in which case there is a straightforward sense in which the former contributes more to scientific progress than the latter.^11^

## Rival accounts of scientific progress

In this section, I consider two of the main rivals to the noetic account of scientific progress, viz. *the truthlikeness account* initially proposed by Popper (1963, 1979) and subsequently developed by Niiniluoto (1980, 1984, 2014, 2017), and *the epistemic account*, as formulated and defended by Bird (2007, 2008, 2016, 2019).^12^ For each account, I will compare it to the noetic account—highlighting the points on which the accounts are in agreement, and explaining where they diverge—and then briefly argue that the noetic account improves on its rival.

### The truthlikeness account

Briefly, the truthlikeness account holds that scientific progress occurs when accepted scientific theories get closer to the truth, i.e. become more truthlike. In the special case of one theory $T_1$ being replaced with another theory $T_2$ (with no other changes or additions to accepted theories), scientific progress occurs if and only if $T_1$ is more truthlike than $T_2$. The key notion of *truthlikeness* (or *verisimilitude*) is meant to measure the extent to which a theory captures the complete truth about the world in a given conceptual framework. Thus, one way in which $T_2$ may be more truthlike than $T_1$ is if $T_2$ makes true (or approximately true) claims on which $T_1$ is silent, since $T_2$ would thus capture a larger part of the complete truth about the world than $T_1$. Another way for $T_2$ to be more truthlike than $T_1$ is if $T_2$ corrects some false claims made by $T_1$. In both cases, replacing $T_1$ with $T_2$ would constitute progress on the truthlikeness account.

In Niiniluoto’s version of the truthlikeness account, which is the most developed account of this sort in the literature, the truthlikeness of a scientific theory *T* is defined relative to a language *L*. Roughly, *T*’s truthlikeness is then a measure of the similarity between a maximally specific claim C* in *L*, which fully captures everything that is true, and a disjunction of other such maximally specific claims $(C_1 \vee ... \vee C_n)$ in *L*, which captures the content of *T* by effectively listing all the maximally specific possible states of affairs allowed by *T* (Niiniluoto 1987; see also Oddie 1986). This definition of truthlikeness brings out a rather notorious problem for truthlikeness accounts, viz. that extant definitions are ‘language-dependent’ in the sense that the truthlikeness of *T* may be higher or lower in another language $L'$ as compared to *L*. In so far as it is implausible that there is any single objectively correct language relative to which truthlikeness can be defined, this leads to progress being language-relative. It is a matter of contention whether this is a serious problem for the truthlikeness account (see, e.g., Miller 2006; Bird 2016; Oddie 2016; Niiniluoto 2017); since this is well-trodden terrain, I will not comment further on this issue here.

In comparing the truthlikeness account to the noetic account, the first thing to say is that the two accounts are similar in two important respects. First, the intuitive notion of truthlikeness (of theories) corresponds quite closely to the noetic account’s two notions of accuracy and comprehensiveness (of dependency models). Thus, were it not for certain connotations of the term ‘truthlikeness’, such as the language-relativity therein and its focus on theories rather than dependency models, it would not be too misleading to state the noetic account in terms of increasing truthlikeness of dependency models.^13^ Second, the truthlikeness account resembles the noetic account in imposing no distinctively epistemic requirements on scientific progress, such as the requirement that progressive theories or models be epistemically justified. Of course, as Niiniluoto (2017, pp. 3299–3300) notes, accepted scientific theories generally enjoy at least some degree of empirical confirmation, but it does not follow on either account that scientific progress cannot occur in the absence of the type of justification required for knowledge (more on this in Sect. 4.2).

Regarding the differences between the noetic and truthlikeness accounts, note that where the truthlikeness account focuses on (increasingly truthlike) *theories*, the noetic account focuses on (increasingly accurate and comprehensive) *dependency models*. The main difference between these is that dependency models target specific phenomena in the world, whereas theories are more general and abstract claims with no particular target.^14^ Of course, the two are not unrelated. As some of my examples above intimate, scientists *use* (or apply) theories to gain understanding of phenomena, i.e. to construct dependency models thereof. Earlier we saw how Hooke’s law, $F_s=-kx$, together with Newton’s second law of motion, $F=ma$, can be used to construct a dependency model of a (hypothetical) system consisting of an elastic spring and an attached object. This model reveals that, and how, the object’s acceleration *a* depends on its mass *m*, the displacement distance *x*, and the spring’s stiffness *k*. Since true or truthlike theories undergird understanding in this way, they are profoundly important for scientific progress from the noetic account’s point of view.

With that said, increasing understanding and increasing truthlikeness can come apart; when they do, progress follows the former rather than the latter. Consider first cases in which already existing theories are used to construct new dependency models that are either more accurate or more comprehensive than previous models. In such cases, theory stands still while understanding marches on. Our simple example of the spring provides a case in point. In constructing the dependency model of the system, with which we see (among other things) what and how the object’s acceleration depends on, we did not increase the truthlikeness of our theories. Admittedly, there is a sense in which a new ‘theory’ was added when we derived $a=-\frac{kx}{m}$ from Hooke’s law and Newton’s second law of motion. However, precisely because this ‘theory’ follows logically from previously accepted theories, and thus adds no logical content to them, it cannot possibly increase the truthlikeness of accepted theories. Thus the truthlikeness theorist is forced to say, implausibly, that there is no progress in cases of this sort.

Another way in which increasing understanding can come apart from increasing truthlikeness concerns the use of idealizations to gain understanding. For our purposes, idealizations can be understood as falsehoods that are deliberately included in some representation. Now, in some cases, accepting theories with idealizations increases the truthlikeness of accepted theories in a straightforward way, since the idealized theory may capture part of the complete truth about the world in a way that previous theories failed to do—even when the new theory contains an idealization and is thus false (Niiniluoto 2017, p. 3298). So to see how the noetic and truthlikeness accounts diverge in this respect, we must look to cases in which idealizations play a role in scientific progress even when more truthlike versions of the relevant theories are, or could be, accepted. Specifically, the cases I have in mind are those where a true or truthlike theory is accepted, and yet a corresponding idealized (and thus less truthlike) theory is either adopted or kept on the books^15^ because the latter facilitates understanding in a way that former fails to do.^16^

To use a familiar example, consider that the standard derivation of Boyle’s law ($P \propto \frac{1}{V}$) assumes that the molecules in a gas never collide with each other. Since this assumption is blatantly false of any real gas, the set of theories used in the derivation of Boyle’s law is clearly less truthlike than an alternative set of theories in which this assumption has been replaced with the (true) assumption that, while the molecules do collide, these collisions balance each other out. Indeed, Boyle’s law can be derived from this set of strictly true theories as well, so the truthlikeness account cannot even claim that the idealization here is a ‘necessary evil’ in our path towards true or truthlike theories. So why is the blatantly false assumption that molecules don’t collide kept on the books at all, as part of the publicly available information that scientists, engineers, and others, can draw upon? Why not throw it out like any other falsehood that has been replaced by a true or more truthlike alternative?

Roughly following Strevens’s (2008, 2017) account of idealization, I suggest that the answer is that the idealization facilitates understanding in a way that the non-idealized assumption does not. The inclusion of such an obvious falsehood—that the molecules don’t collide at all—is a way of highlighting the *absence* of a dependence relation—in this case, between Boyle’s law holding of a particular gas, on the one hand, and whether and the extent to which its molecules collide with each other, on the other hand. Put differently, the idealization conveys in an especially dramatic way that for Boyle’s law to hold of a given gas, it is irrelevant whether collisions occur between the molecules in the gas. This is the type of ‘negative’ information about a phenomenon’s dependence relations that may be involved in understanding the phenomenon (see Sect. 3). Thus, on the noetic account, the derivation of Boyle’s law from idealized assumptions constitutes progress, even when a non-idealizing derivation is also available.

The standard derivation of Boyle’s law is but one example among many in which a set of theories containing an idealization provides more understanding than its de-idealized counterpart. It does this in virtue of revealing something about what the target phenomenon *doesn’t* depend on.^17^ Here’s another example. Derivations of trajectories of planets around stars frequently assume that both the planets and the stars are *point masses*, i.e. extensionless particles with positive masses. Of course, we know that this is not just false, but impossible. This would be a problem if the assumption of point masses was meant to convey ‘positive’ information about what the planets’ trajectories do depend on; however, as a way of conveying ‘negative’ information about what these trajectories do not depend on, the assumption of something impossible serves as an especially vivid way to flag that the trajectories do not depend on the volumes of planets or stars. Thus, while including this idealization—this blatant falsehood—clearly doesn’t increase the truthlikeness of our theories, it does increase our understanding of the planets’ trajectories.

To sum up the discussion so far, then, the noetic account comes apart from the truthlikeness account in at least two ways. On the one hand, the noetic account counts as progressive episodes in which already-accepted theories are *applied* to increase our understanding of specific phenomena. On the other hand, the noetic account also counts as progressive episodes in which *idealizations* are introduced to convey what a target phenomenon does not depend on—even when non-idealized alternatives are available. In both cases, the noetic account expands the range of progressive episodes from what is counted as such by the truthlikeness account.

Are there also episodes that the truthlikeness account counts as progressive but the noetic account doesn’t? Such cases would have to involve increases in the truthlikeness of accepted theories that fail to increase our understanding of relevant phenomena. However, from the noetic account’s point of view, the point of proposing new theories in science is to increase our understanding in one way or another. Consequently, there should be very few if any cases in actual scientific practice of increasingly truthlike theories that fail to increase understanding in one way or another. Even theories that are far removed from empirical reality, such as string theory, contain a lot of information about dependencies (e.g. that a particle’s mass depends on the vibrational state of the corresponding string), and thus potentially provide us with great deal of understanding.

But although cases of increasing truthlikeness without increasing understanding will be rare in scientific practice, we can easily conceive of hypothetical cases. Consider *entirely spurious correlations*: statistical correlations between two or more phenomena that aren’t due to any dependence (e.g. causal) relation between those phenomena, or between these phenomena and some other phenomenon. For example, it is presumably entirely spurious that the average margarine consumption in the U.S. was highly correlated ($r=0.9926$) with divorce rates in the state of Maine in the years 2000–2009 (Vigen 2015, pp. 18–20). The ‘theory’ that these two quantities are correlated is truthlike—indeed, fully true. So if this correlation were to be accepted, it would presumably constitute progress on the truthlikeness account. However, this ‘theory’ arguably couldn’t increase anyone’s understanding of either U.S. margarine consumption or Maine divorce rates, since it fails to tell us anything about what these quantities depend on, e.g. what causes or grounds them. Thus the acceptance of this claim would not constitute progress on the noetic account, regardless of how truthlike it is.

One might worry that the noetic account goes too far in discounting spurious correlations as non-progressive. Does this imply that searching for correlations is never a worthwhile scientific practice? Not at all. Although correlation is not causation—or any kind of dependence relation, for that matter—the former is normally a (fallible) guide to the latter. Thus, correlations often *promote* progress on the noetic account, e.g. through prompting more serious studies of the correlated variables where researchers control for possible confounders. However, this only holds when the correlations in question are not entirely spurious in the above sense, i.e. when the correlation is due to a dependence relationship between those phenomena or between them and a third phenomenon. So, on the noetic account, entirely spurious correlations do not even promote progress in the way that non-spurious correlations normally do, which explains why they seem so frivolous from a scientific point of view.

### The epistemic account

Bird’s (2007, 2016) epistemic account holds that scientific progress occurs precisely when scientists *accumulate knowledge*. The key term ‘knowledge’ is notoriously difficult to define, and Bird agrees with Williamson (2000) that it is unanalyzable and *sui generis*. Regardless, Bird follows epistemological orthodoxy in taking knowledge to require truth, belief, and epistemic justification. That is, one cannot know something unless it is true, one believes it, and one is justified in believing it. Two of these three requirements, viz. truth and belief, have analogues in the noetic and truthlikeness accounts in so far as both require progressive representations to be more accurate/truthlike, and that these representations are, or could be, in some sense accepted, adopted, or grasped by some agents. By contrast, no version of the requirement that progressive theories be epistemically justified is present in either the noetic account or the truthlikeness account.^18^ Thus, although other components of the epistemic account might also be problematic, we shall focus on the justification requirement in what follows.

Before we begin, however, let me clarify that to reject a justification requirement on scientific progress is not tantamount to claiming that the practice of seeking confirmation for scientific claims plays no role in the progress of science. Far from it. The point of scientific confirmation is to separate, as far as possible, fact from fiction. For the noetic account, the relevant facts are those that can be used to construct models of dependence relations, which in turn constitute understanding. Without scientific confirmation, these models would generally be woefully inaccurate, and thus fail to constitute understanding. Moreover, even if by some fluke an unconfirmed model were to be sufficiently accurate to increase our understanding, in the absence of scientific confirmation we would not be able to tell it apart from alternative, inaccurate models. Consequently, an unconfirmed but accurate model would rarely, if ever, in fact be used by any of us to increase our understanding. For these reasons, scientific confirmation certainly plays a key role in the progress of science on the noetic account.

What really separates the epistemic from the noetic account (and from the truthlikeness account)^19^ is whether epistemic justification, i.e. the type of justification that is required for knowledge, partly constitutes scientific progress. According to the epistemic account, a scientific theory or model that fails to be epistemically justified cannot constitutively contribute to scientific progress, because such a theory or model would fail to be known. Indeed, Bird argues for the epistemic account by appealing to actual and hypothetical cases in which scientists form unjustified, but nevertheless true, beliefs about scientific phenomena. In these cases, Bird claims that the epistemic account “accords with the verdict of intuition”, while not requiring justification for progress “conflicts with what we are intuitively inclined to say” (Bird 2007, p. 66). In particular, Bird says that that it would not have been progressive for scientists to accept Alfred Wegener’s theory of continental drift when Wegener first proposed it in 1912, because the theory was not sufficiently justified at the time to count as knowledge. Although Bird targets the truthlikeness account specifically, his argument would apply also to the noetic account if Wegener’s theory would have been made publicly available in a way that made it possible for relevant members of society to increase their understanding of relevant phenomena, e.g. the lithosphere of the Earth.

Many commentators disagree with Bird’s intuitions about such cases (Rowbottom 2008; Cevolani and Tambolo 2013; Niiniluoto 2014; Dellsén 2016).^20^ More importantly, it is unclear why (alleged) facts about what “we are intuitively inclined to say” should count for much at all in discussions of scientific progress. After all, as noted above, the question of scientific progress is unmistakably normative: it is not about the extension of a concept that we happen to possess, but about what types of cognitive changes in science ought to be pursued and incentivized (see Sect. 2). For the purposes of answering the latter type of question, we should arguably consult our *reflective* judgments rather than our untutored intuitions. Of course, the outcome of such reflections might be that we we may end up agreeing with what our previous selves were already inclined to say, i.e. with our original intuitions—but that, too, would be a reflective judgment.

With all this in mind, I turn now to presenting an objection to the claim that justification is necessary for scientific progress—and thus, by implication, to the epistemic account.^21^ This objection appeals to a type of *higher-order evidence*, i.e. evidence about the epistemic character of some other, typically first-order, evidence (Christensen 2010; Kelly 2010). What is interesting about higher-order evidence is that, in many cases, it undermines or defeats the epistemic justification otherwise provided by first-order evidence.^22^ In science, the first-order evidence is simply what we would usually call ‘scientific evidence’, the type of evidence that is systematically collected in science and published in scientific journals (e.g. observational data and experimental results). Thus higher-order evidence in science could potentially undermine or defeat the epistemic justification provided by ordinary, first-order scientific evidence. If so, this type of higher-order evidence in science would, in a roundabout way, prevent progress from occurring according to the epistemic account—even in cases where our theories/models are true/accurate.

Consider a form of higher-order evidence that should be particularly familiar to philosophers of science, viz. historical higher-order evidence. According to a general version of the *pessimistic meta-induction* (e.g., Poincaré 1952; Hesse 1976; Laudan 1981a), most past theories (including many of the most successful ones) have turned out to be false by our current lights; hence, by enumerative induction, we have reason to believe that most of our current theories (including many of the most successful ones) will suffer the same fate. Note that this is an an argument that the supposed historical failures of scientific theories undermine or defeat the epistemic justification for current theories that would otherwise be provided by the ordinary, first-order scientific evidence in their favor. Thus the historical record is, according to the pessimistic meta-induction, a type of higher-order evidence against current theories being epistemically justified. In so far as the pessimistic meta-induction is successful, no such theories would be epistemically justified, regardless of how highly confirmed they are by ordinary first-order scientific evidence, because the historical higher-order evidence would prevent it from providing justification for current theories.

Admittedly, there are reasons to think that this general version of the pessimistic meta-induction greatly overstates the extent to which the historical record undermines the justification for current theories provided by the first-order evidence in their favor. Many of the central posits of past theories are preserved in current theories (e.g., Kitcher 1993; Psillos 1999; Chakravartty 2007), and current theories are arguably better confirmed by first-order scientific evidence than their past counterparts (Roush 2010; Fahrbach 2011, 2017). So it is doubtful, at best, that the historical record supports the *wholesale* conclusion that current scientific theories are epistemically unjustified across the board. With that said, it seems undeniable that, at least in some cases, more *local* versions of the pessimistic meta-induction does indeed undermine the epistemic justification for scientific theories at various points in history (Ruhmkorff 2013; Asay 2019)—including some episodes that are arguably paradigmatic of scientific progress.

Thus consider cases where, in a particular scientific domain or discipline *D*, scientists have in the past successively adopted theories $T_1,...,T_{n-1}$, none of which are even approximately true by our current lights. Consider a point in time where the most recent theory in *D*, $T_n$, has only recently been adopted. Then, even if $T_n$ is at least as well confirmed by the first-order scientific evidence as any of its predecessors, the higher-order evidence against $T_n$ could be sufficiently strong (e.g., because *n* is sufficiently high) to defeat the justification that would otherwise be conferred on $T_n$. Hence $T_n$ cannot, at least not at this point, be known. But is it plausible that this historical fact about the previously adopted theories by itself prevents the adoption of $T_n$ from contributing to scientific progress? Indeed, supposing that $T_n$ is otherwise of the standard required for progress, e.g. in enabling us to increase our understanding of relevant phenomena, then isn’t the adoption of $T_n$ all the more progressive given that previous theories in the same domain *D* were so far off track?

A historical case, familiar from debates about the pessimistic meta-induction (Stanford 2006, pp. 51–140), may be used to illustrate the point. In the latter half of the 19th century, various theories were proposed by the most eminent biologists of the day to explain the mechanism by which biological traits are inherited from one generation to the next. Chief among these were Charles Darwin’s *pangenesis theory*, proposed in 1868; Francis Galton’s *stirp theory*, proposed in 1879; and August Weismann’s *germ-plasm theory*, proposed in 1892. Shortly thereafter, in 1902–1904, Walter Sutton and Theodor Boveri independently developed versions of the currently accepted *chromosome theory*, according to which chromosomes located in all dividing cells carry genetic information from parent to offspring. Assuming that the chromosome theory is indeed correct, the three earlier theories were all fundamentally mistaken, in that each posited some non-existent carrier of genetic material—‘gemmules’ for Darwin, ‘stirps’ for Galton, and ‘germ-plasm’ for Weismann. Now consider a point in time shortly after Sutton and Boveri’s theory was proposed, e.g. 1905. Did their theory contribute to scientific progress at that time?

According to the epistemic account, the answer must be ‘no’. The historical record of failed theorizing about heredity—i.e. the pangenesis, stirp, and germ-plasm theories of Darwin, Galton, and Weismann, respectively—indicated that this most recent theory would suffer the same miserable fate. Even if the first-order scientific evidence in favor of the Sutton-Boveri chromosome theory was already strong at the time, the fact that theorizing in this domain had turned up so many theories that were, by their lights at the time, mistaken, prevents this evidence from epistemically justifying the chromosome theory in the way it otherwise would have. It follows that scientists accepting or believing the theory would not qualify as knowledge, in which case the episode fails to constitute scientific progress on the epistemic account. Thus, whereas we might have thought that Sutton and Boveri’s chromosome theory was all the more progressive in virtue of replacing fundamentally mistaken theories, the epistemic account evidently delivers the opposite verdict that the episode did not constitute progress at all.

The noetic account offers a very different analysis of these types of cases. The chromosome theory accurately depicts the underlying causal mechanism of biological inheritance, which in turn allows us to increase our understanding of, among other things, actual inherited traits (such as the color of your eyes). Thus, as soon as the chromosome theory was made publicly available, primarily via Sutton’s publication of the theory in the recently established *Biological Bulletin* (Sutton 1902, 1903), there was progress on the noetic account. The noetic account does not also require the theory to be epistemically justified in the sense required for knowledge. Consequently, the historical higher-order evidence which serves to undermine or defeat the justification for the chromosome theory does not in any way prevent it from contributing to progress on the noetic account. Hence the noetic account, in contrast to the epistemic account, straightforwardly counts this and similar episodes as constituting scientific progress.

To reiterate an earlier point, this does not imply that scientific evidence or confirmation is of no relevance to scientific progress on the noetic account. If the chromosome theory had not been supported by (first-order) scientific evidence, such as Boveri’s experiments with sea urchins and Sutton’s work on grasshoppers, the theory would probably not have been published at all, and it would certainly not have achieved the status that it later did. In this way, ordinary first-order scientific evidence is crucial to scientific progress on noetic account. What is not crucial—indeed, irrelevant—is whether there is some historical higher-order evidence available which would prevent this first-order scientific evidence from providing the type of epistemic justification for scientists’ beliefs in the chromosome theory that would make them constitute knowledge.

## Challenges to the noetic account

In this section, I consider several challenges to the noetic account that aim to show that it is too narrow to accommodate the full range of cases that plausibly fall under scientific progress. The general worry here is that by identifying scientific progress with enabling increased understanding, rather than with some more general developments such as increased truthlikeness of accepted theories, we have excluded a variety of developments that ought to count as progressive. I will consider three specific versions of this worry, viz. that the noetic account is too narrow in virtue of (i) making metaphysical assumptions regarding dependence relations, (ii) excluding scientifically important classification schemes, and (iii) excluding discoveries of previously unknown phenomena. My contention will be that, ultimately, none of these charges hit home because the noetic account is not as narrow as one might have thought.

Let me first acknowledge, however, that there are two alternative strategies for responding to these challenges. The first is to concede that the noetic account is too narrow as it stands, and subsequently modify the account so as to incorporate other developments than those that enable increased understanding. There are many ways to do this. Most straightforwardly, one might combine elements of the noetic account with elements of alternative accounts, such as the truthlikeness account—and say, for example, that progress consists in enabling increased understanding *or increased truthlikeness*.^23^ The obvious downside to this hybridization strategy is that it sacrifices the simplicity of the (non-hybird) noetic account. Ultimately, one may of course end up thinking that this is a sacrifice worth making. But we won’t know until we have thoroughly considered whether the noetic account is able to respond convincingly to the challenges (i)–(iii) without hybridization. So let’s see how far we can get with the (non-hybird) noetic account before we concede ground to its opponents and adopt a hybrid account instead.

A second alternative strategy is a revisionist one. Faced with the charge that the noetic account is too narrow, e.g. in virtue of counting classification schemes as non-progressive, one could argue that its narrowness is a virtue rather than a vice. The narrowness of an account of scientific progress is what gives it its critical bite—its potential for serving as the basis of philosophically informed decisions about which research projects to pursue (at all, or at the expense of others). Note, for example, that an account of scientific progress that accommodates all scientific developments as progressive can’t ever deliver the verdict that some projects are not worth pursuing at all, which is one of the purposes to which such an account would be put.^24^ So, in some instances, the correct response to charges of narrowness might be to embrace it as a desirable feature of accounts of scientific progress. Although this type of revisionist strategy is indeed appropriate for some purported cases of scientific progress (see Sects. 5.2 and 5.3) I do not think it works as a general strategy since many of the apparently-excluded developments are very much worth pursuing.

### Excess metaphysical baggage?

The first challenge that I will consider is that the noetic account appears to assume, in a way that the truthlikeness and epistemic accounts do not, that there are certain metaphysical relations in the world, e.g. causation and grounding, which our dependency models come to accurately represent to some degree in cases of scientific progress. But what if the world is metaphysically sparse, devoid of necessary connections between distinct existences, as per Hume’s dictum (Wilson 2010)? What if the things we call ‘causation’ and ‘grounding’ are mere shadows of reality, e.g. regularities that we happen to notice in our experiences? If so, it might seem as if the noetic account would make scientific progress not just rare, but impossible. After all, there would be no dependence relations out there in the world for us to represent in such a way as to make scientific progress possible on the noetic account.

The short response to this challenge is that, appearances perhaps to the contrary, the noetic account is compatible with metaphysical outlooks that entirely reject necessary connections in nature. All that’s required for understanding is that there be some facts of the matter about how one thing depends on (e.g. is caused by) another. It does not matter whether these facts of the matter are ultimately facts about the fundamental fabric of reality, or whether they are instead reducible to or explained by other features of reality, such as regularities in our experiences, our human psychology, or our social practices. Thus, for example, one can easily pair the noetic account with a regularity theory of causation, such as Mackie’s (1974), on which causal relations are nothing over and above certain regularities in the events constituting the purported causes and effects.^25^ Since such theories do not deny that—indeed explain how—some events cause others, they clearly don’t make it impossible to accurately represent causal relations.

As far as grounding is concerned, the situation is essentially similar although slightly more delicate. A complication comes from the fact that some authors use the term ‘grounding’ in a way that prejudges metaphysical questions, e.g. about the independent existence and fundamentality of the grounding relation itself, or about the grounding entity being more fundamental than what it grounds (e.g., Schaffer 2009; Raven 2016). For the purposes of this paper, I don’t mean for the notion of ‘grounding’ to carry any such metaphysical baggage. Rather, my use of the term is merely meant to refer to a type of non-synchronic relation that is analogous to causation, and that typically holds between a reduced object, state, or property, on the one hand, and its reductive base, on the other hand. Without some such notion, it seems to me that it would be hard to make sense of the way in which we understand the properties of *water* by reducing it to $H_2O$, for example. An accurate and relatively comprehensive dependency model ought to reflect the ways in which the various observable properties of water, e.g. its being liquid at room temperature, depend on its underlying chemical composition (and not vice versa).

But while we thus arguably need something like the notion of ‘ground’ to account for some types of understanding in science, we don’t need to make any metaphysically loaded assumptions about what it refers to (Dasgupta 2017). In particular, we need not posit the existence of any fundamental, primitive, or unified relation in the world to which the notion refers. Instead we can agree with ‘grounding skeptics’, who argue that grounding is to be identified with or reduced to other metaphysical dependence relations, such as type or token identity, supervenience, or determination (Wilson 2014; Koslicki 2015; Hofweber 2016), which may or may not themselves be reducible to something less metaphysically bloated. Alternatively, grounding may well turn out to be a form of non-diachronic causation (Wilson 2018), in which case reductive theories of the latter could arguably be applied to the former as well. Furthermore, a possibility left open by the noetic account is that the dependence relations normally called ‘grounding’ are largely due to mind-dependent psychological facts about what human beings happen to classify as explanatory rather than any sort of fundamental facts about reality (Norton and Miller 2019). In any case, it should be clear that the notion of ‘grounding’ to which I have cautiously appealed above carries no special metaphysical baggage beyond what is already needed to account for commonplace scientific reductions such as that between *water* and $H_2O$.

### Non-progressive classification schemes?

Another challenge for the noetic account concerns classification schemes used in science, such as the periodic table of elements and the Linnaean system of biological classification. The challenge here is that, in contrast to ordinary physical theories, for example, it is less clear what information about dependence relations is conveyed by such classification schemes. Indeed, one might argue that in so far as such schemes tell us anything, they merely describe various properties of the classified entities in a particularly economical manner without ever taking a stand on the causes or grounds of these entities or their properties. So does the noetic account imply that developing classification schemes contributes nothing towards scientific progress?

I think not. To see why, let us start by noting that no plausible account of scientific progress should count *all* classification schemes as contributing to scientific progress. The purpose of any classification is to convey information in an efficient manner (Mill 1874; Mayr 1974). Indeed, all classification schemes convey some information or other—minimally, they convey that the elements in a given category satisfy the conditions for membership of that category. So the question is, what type of information must a given classification scheme convey in order for its adoption to count as progressive? Here different accounts of scientific progress clearly part ways, in so far as they count different types of information as progressive. Let us focus on the noetic account, against which the current challenge is directed. This account implies that progress-constituting classification schemes convey information about dependence relations, e.g. causal relations, that might hold between the classified entities or between those entities and other entities not classified in that scheme. In addition, the noetic account also envisions progress-promoting classification schemes, which would roughly be those that cause or raise the probability of enabling increased understanding at some later time.

In my view, it’s plausible that these are precisely the types of classification schemes that are found to be of value in scientific practice. To substantiate this claim, consider first the information contained in the periodic table of elements (see Scerri 2007). The classification of certain elements into groups serves to highlight the ways in which these elements’ atomic structure is responsible for their distinctive macro-level properties. For example, the periodic table nicely conveys the information that the six naturally occurring elements classified as ‘noble gases’ have similar chemical properties (e.g., being odorless, colorless, and generally unreactive) due to to having a similar atomic structure (viz., a full outer shell of valence electrons). Indeed, it is in virtue of latching onto dependence relations of this type that the periodic table enjoys such remarkable predictive success that Mendeleev was able to use it to predict the discovery of previously unknown elements with pre-specified chemical properties (Scerri and Worrall 2001). Far from counting the periodic table as non-progressive, then, the noetic account explains the value of the periodic table as conveying exactly the type of information that serves to increase understanding.

Let us also consider the Linnaean system of biological taxonomy, which classifies biological species hierarchically into higher taxa at different ranks (primarily *genus*, *family*, *order*, *class*, *phylum*, and *kingdom*). Any discussion of this system is complicated by the fact that there is not agreement among biologists about which species should be grouped together at each rank (see Hull 1988, pp. 158–276). The most widely accepted view, *cladism* (e.g., Hennig 1966), holds that biological classification should be based on recency of common descent and thus reflect the evolutionary relationships between different species. So if two species evolved from a common ancestor, from which a third species did not evolve, then cladism implies that the two aforementioned species should at some rank be classified together in a way that excludes the third. For example, birds and crocodiles share a common ancestor that is not an ancestor of lizards, so cladism implies that birds and crocodiles should at some rank be grouped together in way that excludes lizards (Sober 2000, pp. 165–166). Since a cladistic classification scheme is thus explicitly designed to reflect causal relationships between (current and past) species, it conveys understanding in a straightforward manner. Thus the development of a cladistic taxonomy clearly counts as progressive on the noetic account.

What if cladism is rejected, despite its popularity? Even if we think cladism is correct, we may want our account of scientific progress to be consistent not just with our preferred view of biological classification, but also with other views that are taken seriously by working biologists. Here I cannot consider all alternatives to cladism, but let me nevertheless briefly consider the alternative that stands in starkest contrast with cladism, viz. *phenetics* (e.g., Sneath and Sokal 1973). In a phenetic taxonomy, species are grouped together in higher taxa based on ‘overall similarity’, regardless of how they are evolutionarily related. For example, since lizards and crocodiles are arguably more similar to each other than either of them is to birds, pheneticists typically hold that lizards and crocodiles should be grouped together in a way that excludes birds. The underlying idea behind phenetics is that ‘overall similarity’, e.g. in observable traits, is a more objective or theory-neutral basis for biological classification than evolutionary ancestry. This might seem to go against the noetic account, in so far as phenetic taxonomies fail to directly convey any information about causal relationships between species.^26^

However, things will not seem so straightforward once we consider the main motivation for developing phenetic taxonomies. Prominent pheneticists, such as Sneath and Sokal (1973), were motivated not by a desire to avoid causal relationships between species in biological theorizing. On the contrary, they maintained that a phenetic taxonomy would be better suited than a cladistic one as a theoretically neutral basis for making inferences about evolutionary relationships. On the pheneticists’ view, developing a cladistic taxonomy risks begging the very question that a biological classification scheme ought to help us answer, viz. how different species are evolutionarily related. So the phenetic point of view is that biological classification should contain the data from which evolutionary relationships are inferred, as opposed to containing the conclusions of such inferences (see Hull 1988, pp. 117–120). Put differently, the main point of a phenetic taxonomy is to *promote* the discovery of evolutionary relationships, which are causal relations. So while developing a phenetic taxonomy would admittedly not constitute much scientific progress on the noetic account, it would certainly—indeed, is specifically designed to—promote progress.^27^

To sum up, then, the noetic account provides a framework for making sense of the debate from both sides of the cladism-phenetics divide. Cladists hold that biological classification ought to reflect the underlying causal relationships between species, so that a taxonomy directly conveys information that increases our understanding of biological species. Successfully developing cladistic classification schemes therefore constitutes scientific progress on the noetic account. By contrast, pheneticists hold that biological classification ought to reflect the current ‘overall similarities’ between species, regardless of ancestry. But the point of such classification is to help evaluate, in a supposedly theory-neutral way, hypotheses about the causal relationships between species. Successfully developing phenetic classification schemes therefore promotes scientific progress on the noetic account. Either way, the noetic account can effortlessly explain the scientific value of biological taxonomies.

With all of that said, there will of course be some—indeed, infinitely many—classification schemes that the noetic account counts as more-or-less worthless as far as scientific progress is concerned, i.e. as neither constituting nor promoting any noteworthy degree of progress. If the noetic account is correct, these will inevitably be a bit silly. For example, consider a classification of all objects in the universe into those that are less than 10 m from the tip of my nose in any direction, and those that are outside of this sphere. Presumably, this classification conveys little or no information about dependence relations, and promotes little or no discoveries of them either. Hence it counts as relatively useless for the purposes of scientific progress on the noetic account (and rightly so). Generally, then, whether a given classification scheme counts as constituting or promoting progress, or as doing neither, depends on the classification scheme in question, and the use to which it is put. So, on the noetic account, the relationship between progress and classification will have to be evaluated on a case-by-case basis. I hope it’s clear, however, that the noetic account does plausibly count as progressive two of the most prominent classification schemes in current science, viz. the periodic table and Linnaean taxonomy.

### Non-progressive existential discoveries?

I turn now to a final challenge to the noetic account. Roughly, the challenge is to accommodate discoveries of new phenomena, such as previously unknown biological species, new physical effects, and archeological findings. The worry is that such discoveries might not enable anyone to increase their understanding since they don’t necessarily contain information about dependence relations. A closely related worry is that the noetic account might not count theoretical postulations of (real) entities as progressive, again because the mere posit that an entity exists doesn’t necessarily contain information about dependence relations. What unifies these worries is the concern that the noetic account does not account for progress through what we may call *existential discoveries*, viz. empirical or theoretical uncoverings of previously unknown entities.^28^

The first thing to note about this challenge is that it is clearly not the case that all existential discoveries are scientifically progressive—or, if they are, some are much less progressive than others. Bird (2007) imagines researchers who count, measure, and classify billions of grains of sand on a particular beach. As Bird admits, this “adds little to scientific progress” (Bird 2007, p. 84). So, *a fortiori*, had the researchers ‘discovered’ only a particular grain of sand, this adds even less—if indeed anything at all—to scientific progress. To take an even more extreme example, consider Charles Dawson’s discovery in 1921 of the skull fragments that became known as the ‘Piltdown man’. The composition of these fragments, with canine teeth but a human-like skull, suggested that they came from an early humanoid that might serve as the ‘missing link’ in the evolution of humans from other primates. However, this discovery was not progressive (indeed, perhaps significantly regressive or progress-demoting) since the skull fragments turned out to be have been fraudulently put together in an effort to deceive archeologists—probably by Dawson himself (Groote et al. 2016). An account of scientific progress that treats ‘discoveries’ like this as on a par with the discoveries of, for example, quarks and platypuses, would clearly be inadequate. So the challenge for the noetic account, or indeed for any account of scientific progress, is not to show how every existential discovery adds (significantly, or at all) to scientific progress; rather, it is to show how some select group of existential discoveries do so and that others don’t (or not as much).

So what would make an existential discovery progressive according to the noetic account? Well, first of all, the discovery of a new entity often directly conveys information about dependence relations. For example, the postulation and subsequent detection of the up and down quarks directly increased our understanding of neutrons and protons, because the latter are constituted by, and thus depend on, the former. This is a case in which the discovered entities (up and down quarks) stand in a dependence relation to already known entities (neutrons and protons) that we are hoping to understand better. There are also cases in which the discovery of an entity indirectly reveals something about dependence relations between other entities. For example, the discovery of the platypus, the first egg-laying mammal to be discovered by Europeans, revealed (to Europeans) that the distinctively mammalian properties of having mammary glands and fur/hair, for example, are not caused by the same speciation event as those that cause most mammals to give birth to live offspring. Put differently, the discovery of the platypus conveys information about the evolutionary lineage of mammals, which of course is a type of information about dependence relations between mammalian species and their ancestral species.

In these examples, existential discoveries convey information about dependence relations, and thus constitute scientific progress on the noetic account. In other cases, such discoveries only or primarily contribute to progress by promoting its occurrence at a later time. The most obvious, and perhaps most common, way in which they might do so is through being *evidence* for claims about dependence relations which in turn increase our understanding. For example, consider Brownian motion, the random fluctuation of particles suspended in liquids or gases, which was discovered already in 1827 by the botanist Robert Brown. Since Brown merely observed the phenomenon, and did not explain it in any way, his discovery conveyed no understanding at the time, and thus didn’t constitute progress on the noetic account. However, Brown’s discovery promoted progress in so far as it caused Albert Einstein (1956) to provide an elegant explanation of Brownian motion based on the kinetic theory of heat and the atomic theory of matter. Thus the discovery of Brownian motion promoted progress, not just on Brownian motion itself but also on the nature of heat and matter, in so far as Brownian motion served as evidence for the kinetic and atomic theories of these respective phenomena.

Finally, even when existential discoveries do not constitute progress by conveying information about dependence relations, and even when they don’t promote progress through being evidence for claims about dependence relations, there is still a third way in which existential discoveries may facilitate progress on the noetic account. Obviously, one cannot understand something that hasn’t been discovered. So when we discover an entity or phenomenon *X*, we are always *enabling* progress with regard to *X* on the noetic account (where ‘enabling’ is a special case of promotion).^29^ Consider, for example, the common but poorly-understood disease variously known as myalgic encephalomyelitis (ME) or chronic fatigue syndrome (CFS). Although the underlying causes of ME/CFS are still quite unclear, its status as a distinct disease has been widely acknowledged in recent years, e.g. by the Center for Disease Control and Prevention (CDC) in the United States (Fukuda 1994). This recent discovery—or, if you prefer, postulation—of ME/CFS is a prerequisite for an understanding of the disease, e.g. through research into its possible neurological and epidemiological causes.^30^

I thus conclude that existential discoveries may count as progressive in three distinct ways. Many such discoveries, e.g. of the up and down quarks, constitute progress, since they reveal information about what other more familiar phenomena, e.g. neutrons and protons, depend on. Other existential discoveries primarily serve to promote progress through constituting evidence for claims about dependence relations, e.g. in the way that Brownian motion led to our current understanding of heat and matter. Finally, all existential discoveries enable progress on the discovered phenomenon itself; thus, in so far as we care to make progress on that phenomenon, such discoveries automatically promote progress.

## Conclusion

*What is scientific progress?* In this paper, I have sought to address this question in two ways. On the one hand, I have precisified the question itself by introducing various distinctions, such as that between constituting and promoting progress, and between progress-on-*X* and overall progress. Thus precisified, I have suggested that the most fundamental question of scientific progress concerns what type of cognitive change with respect to a topic *X* constitutes a scientific improvement (to a greater or lesser extent) with respect to *X*. On the other hand, I have advanced and defended a revised version of the noetic account of scientific progress. A cognitive change constitutes a scientific improvement on *X* just in case it makes scientific results publicly available so as to enable relevant members of society, including scientists themselves, to increase their understanding of *X*. I have sought to show how this account can explain various features of scientific practice that are puzzling or inexplicable on alternative accounts, such as why idealized theories are not always abandoned when more accurate alternative become available, why discovering entirely spurious correlations plays a minimal role in scientific practice, and why higher-order evidence (e.g. from pessimistic meta-inductions) is not an obstacle to scientific progress. Finally, I have defended the noetic account against several challenges that accuse the noetic account of being too narrow to accommodate the full range of cases of scientific progress.

## Footnotes

1 This type of philosophical methodology dates back to Carnap (1962), but has recently been revived under various labels such as ‘conceptual engineering’ (Cappelen 2018), ‘conceptual ethics’ (Burgess and Plunkett 2013a, b), and ‘ameliorative analysis’ (Haslanger 2013).

2 Hence, one can test for whether (one judges that) a given scientific improvement constitutes or promotes progress with a thought experiment in which one imagines that the episode in question either has no effects whatsoever or that its effects clearly do not constitute progress. If the episode is still an improvement, it constitutes progress; if not, it promotes progress.

3 See Rowbottom (2015, p. 104) for another type of example of something that could promote progress without constituting it in certain circumstances, viz. flatly false beliefs that lead to future progress.

4 Consequently, an episode may also promote more and less progress, corresponding to how much progress it leads to or probabilifies. The latter can be measured as the probability-weighted average of degrees of progress in all epistemically possible scenarios, mirroring the definition of expected utility in standard decision theory.

5 For example, Bird (2007, p. 84) explicitly declines to give an account of degrees (or ‘rates’) of progress on the grounds that “it is a much more difficult question” than what determines outright progress.

6 In my view, the most plausible approach to aggregating progress on different topics in this way into a notion of overall progress would take the weighted sum of progress on each topic, where the weights assigned to progress on each topic are determined by the scientific significance, in Kitcher’s (2001, 2011) sense, of the topics in question. Thus if making progress on the evolution of human beings is of greater scientific significance than making progress on the evolution of sea urchins, for example, then the former would contribute more to overall progress than the latter.

7 For arguments that it is superior, see Dellsén (2020). Rival accounts are provided by, among others, Strevens (2013), Wilkenfeld (2013), Grimm (2014), Bengson (2015), Elgin (2017), de Regt (2017), and Khalifa (2017), although many of these accounts have important similarities with mine.

8 That said, what I am calling causation and grounding may well be species of the same genus (Wilson 2018).

9 Some earlier discussions of scientific progress (e.g., Bird 2007; Dellsén 2016) appear to assume that progress is determined by changes in the attitudes of individual scientists. More recently, Bird (2019) (see also Ross 2020 and Harris 2021) has argued that progress is determined by the collective attitudes of scientific communities, where the latter are not neatly reducible to individual attitudes. Relatedly, Niiniluoto (2017, p. 2399) refers to it as a “hidden assumption” that “the primary application of the notion of scientific progress concerns successive theories which have been accepted by the scientific community”.

10 A great deal more could be said about what constitutes ‘publicly available scientific information’, who counts as ‘relevant members’ of society, and how the relevant information ‘makes it possible’ for them to increase their understanding. However, since nothing in what follows depends on how the noetic account is spelled out in these respects, I’ll leave that for another occasion (see Dellsén ms).

11 Indeed, we might even want to say that the former is, in some general sense, “more progressive” than the latter. I have no quarrel with this way of speaking as long as we then keep firmly in mind that accounts of scientific progress (including the noetic account) are not currently meant to directly explicate this general sense of the term.

12 Given the limited space of a journal article, my discussions of these accounts will inevitably be quite brief. I also lack the space to discuss *the functional account* (Kuhn 1970; Laudan 1977, 1981b; Shan 2019), non-standard versions of the truthlikeness and epistemic accounts (e.g., Aronson et al. 1994; Barnes 1991; Northcott 2013; Park 2017), hybrid accounts (e.g., Bangu 2015; Goebel 2019), and various other accounts that have been proposed (e.g., Douglas 2014; Rowbottom 2019; Saatsi 2019).

13 This might seem to suggest that the noetic account comes close to characterizing scientific progress as increasing *legisimilitude*, i.e. closeness to (true) laws of nature (see, e.g., Cohen 1980; Niiniluoto 1983). However, I would resist the characterization of dependence relations as laws of nature, since (i) many dependence relations are much too specific and fragile to count as laws (see Woodward 2003, pp. 239–314), and (ii) some of the relevant dependence relations may be mathematical or logical rather than nomological (see Baron et al. 2017).

14 On many accounts of scientific modelling, this is a general difference between models and theories. See, e.g., Cartwright (1983), Giere (1988), Bailer-Jones (2013), and Weisberg (2013).

15 In the latter case, keeping the idealized theory would not strictly speaking constitute progress so much as the alternative course of action, i.e. discarding the theory, would constitute regress. For the sake of simplicity, I have presented accounts of scientific progress as focusing on progressive episodes, but such accounts must also account for the opposite of progress, i.e. regress, and the lack of either progress or regress, i.e. what we might call ‘flatlining’.

16 In Weisberg’s (2007, p. 642) terminology, these fall under ‘minimalist idealizations’.

17 This is not to say that idealizations are the *only* way to convey information about what a target phenomenon doesn’t depend on. It may just be a particularly efficient way of doing so, especially in cases where one is also seeking to convey information about what the target phenomenon *does* depend on (and how exactly it depends on those factors).

18 Note that the notion of understanding with which the noetic account operates (see Sect. 3.1) does not require epistemic justification. This is in line with arguments that understanding differs from knowledge in this respect (Hills 2016; Dellsén 2017).

19 I will focus on contrasting the epistemic and noetic accounts in what follows, but my criticism of the epistemic account should also be congenial to those who favor the truthlikeness account.

20 Mizrahi and Buckwalter (2014) investigated laypeople’s intuitions about the relationship between progress and justification. As Rowbottom (2015, p. 103) points out, the study appears to go against Bird’s contention the intuitive concept of scientific progress *requires* justification.

21 See also Dellsén (forthcoming), in which this objection is discussed alongside two other objections to the epistemic account’s justification requirement on scientific progress.

22 The type of higher-order evidence most widely discussed in epistemology is (recognized) *peer disagreement*, i.e. situations in which one becomes aware that someone who is equally competent and equally well informed about some issue has formed a contrary belief to one’s own (see, e.g., Christensen 2007).

23 Although this would require one to find a way to exclude the increases in truthlikeness that I have argued are non-progressive, such as spurious correlations (see Sect. 4.1).

24 As emphasized in Sect. 2, accounts of scientific progress are meant to help us *evaluate* rather than merely *describe* scientific developments.

25 Alternatively, one may adopt an agency theory of causation along the lines of Menzies and Price (1993), according to which causation is ultimately a ‘secondary quality’, due in part to (non-causal) features of the world and in part to (non-causal) features of ourselves.

26 For the sake of the argument below, I will assume that phenetic taxonomies do not directly convey any information about causal relationships. In fact, however, one could argue that most if not all such taxonomies do contain causal information, e.g. in that the species that are grouped together at some rank will have similar causal properties. Although this would be consistent with my argument below, I will not pursue this line of defense since I think the main purpose of a phenetic taxonomy is to help us get at the very same type of causal information that cladistic taxonomies aim to describe.

27 Recall that to count a development as promoting rather than constituting progress is not necessarily to downgrade its overall importance for scientific progress, since a merely progress-promoting development might lead to more progress than a progress-constituting development constitutes.

28 It is worth noting that rival accounts of scientific progress, such as the truthlikeness and epistemic accounts, do not seem to have any trouble counting existential discoveries as progressive. After all, the addition of a sufficiently truthlike, and/or known, existential statement would presumably increase the overall truthlikeness of accepted theories, and/or add to the stock of accumulated knowledge.

29 More precisely, ‘enabling’ may be thought of as a subspecies of promotion that makes progress more probable by raising its probability up from 0; whereas promoting generally can raise the probability of progress up from any probability less than 1. (An alternative approach is to sharply distinguish enabling conditions from causes (see, e.g., Lombard 1990), in which case enabling progress might be thought of as distinct from promoting progress.)

30 Does this mean that all existential discoveries enable scientific progress on the noetic account (or indeed on any account)? Not quite. It is true that, for any phenomenon *X*, the discovery of *X* enables progress on *X*. However, it does not follow that the discovery of *X* is overall progressive, since we might place no significance whatsoever on making progress-on-*X*. (See the discussion of Kitcher’s notion of significance in footnote 6.) This is why, I submit, we wouldn’t count the discovery of a random grain of sand on a beach as adding much to progress, not even in the sense of merely enabling progress. For although such a discovery does make it possible to make progress on that particular grain of sand, we simply don’t place any significance on making progress on such a trivial phenomenon.
